# Vascular and Inflammatory Response Patterns Across Fixed-Sequence Handgrip Exercise Conditions in Hemodialysis Patients: An Exploratory Clinical Study with Complementary Experimental Findings

**DOI:** 10.3390/life16101671

**Published:** 2026-10-07

**Authors:** Misa Miura, Mayura Shimura, Naoki Morito, Osamu Ito, Chie Saito, Satoshi Sakai, Kunihiro Yamagata, Masahiro Kohzuki, Yo Hirayama

**Affiliations:** 1 Faculty of Health Sciences, Tsukuba University of Technology, Tsukuba 305-8521, Japan; mshimura@k.tsukuba-tech.ac.jp (M.S.); ssakai@k.tsukuba-tech.ac.jp (S.S.); 2 Department of Nephrology, Faculty of Medicine, University of Tsukuba, Tsukuba 305-8575, Japan; morito@md.tsukuba.ac.jp (N.M.); chie.saito@md.tsukuba.ac.jp (C.S.); k-yamaga@md.tsukuba.ac.jp (K.Y.); 3 Department of Rehabilitation Medicine, Tohoku Medical and Pharmaceutical University, Sendai 981-8558, Japan; oito@hosp.tohoku-mpu.ac.jp; 4 Yamagata Prefectural University of Health Sciences, Yamagata 990-2212, Japan; makohzuki@gmail.com; 5 Hirayama Hospital, Chiba 262-0046, Japan; yoh2405@gmail.com

**Keywords:** hemodialysis, handgrip exercise, endothelial function, exercise intensity, inflammation

## Abstract

Patients undergoing hemodialysis (HD) frequently exhibit endothelial dysfunction and limited exercise capacity, but vascular and inflammatory responses to different handgrip exercise intensities remain unclear. Male Wistar rats with or without chronic kidney disease (CKD) underwent 4 weeks of forelimb exercise or no exercise (n = 5/group). In the clinical study, eight HD and eight non-HD participants completed fixed-sequence 2-week handgrip exercise conditions at 40%, 60%, and 80% of maximal voluntary contraction (MVC), separated by 2-week washouts. Peripheral arterial tonometry (PAT), inflammatory markers, and muscle strength were assessed. In rats, CKD was associated with lower body weight and spontaneous activity; exercise was not associated with significant differences in systolic blood pressure, serum creatinine trajectories, or inflammatory cytokines. Clinically, PAT showed a significant group-by-intensity interaction (*p* = 0.0035), with the largest mean numerical increase from rest in HD after 60% MVC. Tumor necrosis factor-α (TNF-α) showed a significant interaction (*p* < 0.001), whereas interleukin-6 (IL-6) did not (*p* = 0.396). Muscle strength showed no consistent changes across exercise-intensity conditions. Heterogeneous vascular and inflammatory response patterns were observed across the fixed-sequence handgrip exercise conditions in HD. The non-HD participants were not age-matched to the HD group and were therefore treated as a reference group. These exploratory findings should not be interpreted as demonstrating a causal intensity-dependent effect and warrant confirmation in randomized studies.

## 1. Introduction

Patients undergoing maintenance hemodialysis (HD) experience a markedly increased burden of cardiovascular disease (CVD), physical inactivity, and functional decline. Endothelial dysfunction is an important component of cardiovascular risk in chronic kidney disease (CKD) and may develop before end-stage kidney disease and progress with declining kidney function [1,2]. Chronic inflammation, oxidative stress, uremic conditions, and reduced physical activity may further contribute to impaired vascular homeostasis in patients undergoing HD [1,3,4]. Accordingly, strategies that can preserve or improve vascular function while remaining feasible for patients with limited exercise capacity are clinically relevant.

Exercise is an important non-pharmacological intervention for patients with CKD and HD. Aerobic and resistance exercise have been associated with improvements in physical function, cardiovascular health, and quality of life [2,5,6,7], and exercise-induced increases in blood flow and shear stress may promote endothelial nitric oxide bioavailability and vascular adaptation [8,9]. Nevertheless, implementation of conventional whole-body exercise may be challenging in some patients undergoing HD because of reduced exercise tolerance, frailty, treatment-related time constraints, and the practical requirements of exercise programs. Therefore, simple exercise modalities that can be performed with limited space and equipment may provide an additional option for incorporating physical activity into dialysis care.

Handgrip exercise is a localized resistance exercise that requires minimal equipment and can be performed in a variety of clinical or home settings [10]. Its practical characteristics may make it suitable for adaptation to patients undergoing HD. Previous studies have suggested that repeated handgrip exercise can modify peripheral vascular function, potentially through repeated increases in local blood flow and shear stress [9,11]. However, the vascular response to handgrip exercise may depend on exercise intensity. Increasing contraction intensity does not necessarily produce a proportional vascular benefit, as local blood flow, shear stress, and vascular responses may vary according to contraction intensity [12]. Thus, identifying how vascular responses vary across handgrip exercise intensities may be particularly relevant in HD, a population characterized by underlying vascular dysfunction [1,3,7].

Exercise intensity may also influence inflammatory responses. Interleukin-6 (IL-6) and tumor necrosis factor-α (TNF-α) are relevant to the chronic inflammatory milieu of CKD and HD, whereas exercise can induce complex and context-dependent cytokine responses [13,14]. These responses may vary according to exercise mode, intensity, duration, underlying disease, and timing of measurement [14]. Consequently, assuming a uniform anti-inflammatory effect of handgrip exercise may oversimplify its biological effects. Despite the potential clinical utility of handgrip exercise, relatively little is known about whether vascular and inflammatory responses to different handgrip exercise intensities differ between patients undergoing HD and individuals not undergoing HD.

Experimental studies can provide complementary information regarding the physiological response to repeated localized exercise in the setting of CKD. Evaluation of functional responses together with body weight, spontaneous activity, blood pressure, renal function, inflammatory markers, and renal histopathology may help characterize the biological context in which localized exercise is performed. However, such experimental observations should be considered complementary rather than direct evidence of clinical efficacy.

Therefore, the present study combined an experimental study in a 5/6-nephrectomized rat model with a prospective, fixed-sequence, repeated-measures clinical study in patients undergoing HD and non-HD participants. The experimental component evaluated physiological, functional, renal, and inflammatory findings associated with 4 weeks of forelimb exercise and was intended to provide complementary experimental context rather than mechanistic validation of the clinical findings. Because the animal and clinical protocols differed in exercise modality, outcome measures, and study design, the two components were interpreted separately. The clinical component examined vascular and inflammatory responses to handgrip exercise performed at 40%, 60%, and 80% of maximal voluntary contraction (MVC). The primary clinical focus was the pattern of peripheral arterial tonometry (PAT) responses across exercise-intensity conditions, with inflammatory markers and muscle strength evaluated as secondary outcomes. We hypothesized that vascular and inflammatory response patterns would vary across the three handgrip exercise-intensity conditions and may differ between patients undergoing HD and non-HD participants.

## 2. Materials and Methods

### 2.1. Overall Study Design

This study consisted of two complementary components: (1) an experimental study evaluating the physiological, functional, renal, and inflammatory findings associated with repeated forelimb exercise in rats with and without chronic kidney disease (CKD), and (2) a prospective, fixed-sequence, repeated-measures clinical study evaluating vascular, inflammatory, and physical responses to different intensities of handgrip exercise in patients undergoing hemodialysis (HD) and non-HD participants.

The experimental and clinical components were analyzed separately. The animal experiment was used to provide complementary biological information regarding repeated localized forelimb exercise, whereas the primary clinical focus was the vascular response to different handgrip exercise-intensity conditions. Because the experimental and clinical components differed in exercise protocols, outcome measures, and study design, the animal experiment was not intended to provide mechanistic validation of the clinical findings.

### 2.2. Experimental Study

#### 2.2.1. Animals and Experimental Design

Male Wistar rats were obtained from Japan SLC, Inc. (Hamamatsu, Shizuoka, Japan) at 9 weeks of age. CKD model animals were commercially prepared 5/6-nephrectomized Wistar rats. After arrival, the animals were acclimatized for 1 week. Beginning at 10 weeks of age, animals assigned to the exercise groups underwent forelimb exercise for 4 weeks, and assessments were performed at the completion of the experimental period.

Animals were classified according to CKD status and exercise exposure into four groups (n = 5 per group): non-CKD/non-exercise (non-Ex), non-CKD/exercise (Ex), CKD/non-exercise (non-Ex CKD), and CKD/exercise (Ex CKD).

The experimental protocol was approved by the Animal Experiment Committee of Tsukuba University of Technology (approval no. 4-1; 30 March 2022).

#### 2.2.2. Forelimb Exercise Protocol

Animals assigned to the exercise groups performed repeated forelimb resistance exercise using a grip strength apparatus designed for rats (Muromachi Kikai Co., Ltd., Tokyo, Japan). During the exercise, each animal was held by the tail and allowed to grasp a metal bar with its forelimbs while resistance was applied. The exercise consisted of two sets per day, with a total daily exercise duration of approximately 5 min and was performed 7 days/week for 4 weeks. Animals in the non-exercise groups underwent usual cage activity without the scheduled forelimb exercise intervention.

#### 2.2.3. Body Weight and Spontaneous Physical Activity

Body weight and spontaneous physical activity were assessed at the end of the 4-week experimental period. Body weight was measured using a CL-4502 digital scale (CLEA Japan, Inc., Tokyo, Japan). Spontaneous physical activity was quantified using an SWY-30 running-wheel cage (Melquest Ltd., Toyama, Japan) and expressed as daily wheel counts.

#### 2.2.4. Forelimb Grip Strength

Forelimb grip strength was measured using a grip strength meter designed for rats (Muromachi Kikai Co., Ltd., Tokyo, Japan). Three measurements were obtained for each animal, and the maximum value was used for analysis. Grip strength was expressed in grams (g).

#### 2.2.5. Blood Pressure

Systolic blood pressure (SBP) was measured at the tail using an RMP-6008M blood pressure monitoring system (Nihon Kohden Corporation, Tokyo, Japan) at the end of the 4-week experimental period.

#### 2.2.6. Biochemical and Inflammatory Measurements

Blood samples were collected, and the serum was separated and submitted to the Nagahama Life Science Laboratory, Oriental Yeast Co., Ltd. (Nagahama, Japan), for biochemical analyses. Serum creatinine was measured using an enzymatic method. Serum interleukin-6 (IL-6) and tumor necrosis factor-α (TNF-α) concentrations were measured using a Luminex-based assay according to the laboratory’s standardized analytical procedures.

#### 2.2.7. Renal Histopathology

Kidney tissue was examined histologically using Masson’s trichrome staining. Representative sections from CKD animals with and without forelimb exercise were evaluated qualitatively for histopathological findings.

### 2.3. Clinical Study

#### 2.3.1. Participants and Study Design

Sixteen participants were included in the clinical study: eight patients undergoing maintenance HD and eight participants not undergoing HD. The non-HD participants were unpaid volunteers recruited through posters displayed at the authors’ university. They were not recruited as age- or sex-matched controls for the HD group and were therefore treated as a non-HD reference group in the present study. Maintenance hemodialysis was performed throughout the study period in accordance with the Japanese Society for Dialysis Therapy Clinical Guideline for “Maintenance Hemodialysis: Hemodialysis Prescriptions” [15]. No changes in prescribed medications or dialysis membranes were made during the intervention period. All participants provided written informed consent before enrollment.

Exclusion criteria were severe arrhythmia, decompensated heart failure, uncontrolled hypertension, orthopedic conditions preventing exercise, and inability to provide informed consent. Among the eight HD participants, the mean dialysis vintage was 94.1 ± 16.5 months, and three participants (37.5%) had diabetes mellitus. Three participants (37.5%) were receiving antihyperglycemic medication, and four (50.0%) were receiving antihypertensive medication.

The clinical component was a prospective, fixed-sequence, repeated-measures intervention study. Following a resting baseline assessment, participants completed three 2-week handgrip exercise conditions at 40%, 60%, and 80% of maximal voluntary contraction (MVC) in this fixed sequence. A 2-week washout period was included between successive exercise-intensity conditions.

Before the main clinical study, a preliminary study was conducted in two non-HD participants to assess feasibility and to determine the washout interval between exercise-intensity conditions. PAT ratio was measured before and after handgrip exercise at each intensity. No consistent persistence of the preceding post-intervention vascular response was observed at the subsequent pre-intervention assessment. Based on these preliminary observations, a 2-week washout period was incorporated between the 40%, 60%, and 80% MVC conditions.

Measurements obtained at the end of the washout periods were not available in the retained analytical dataset.

The study protocol was approved by the Ethics Committee of Tsukuba University of Technology (approval no. H29-10) and registered with the UMIN Clinical Trials Registry (UMIN000034121).

#### 2.3.2. Handgrip Exercise Intervention

Before the intervention, maximal handgrip strength was measured for each participant. Handgrip exercise was performed using an adjustable commercially available handgrip trainer (YOKELLMUX, Hui’an, Quanzhou, China), for which the resistance could be set in kilograms. For each exercise-intensity condition, the resistance was individually adjusted to correspond to 40%, 60%, or 80% of the participant’s measured maximal handgrip strength. These target levels are referred to as 40%, 60%, and 80% MVC throughout the manuscript.

The exercise-intensity conditions were performed in a fixed sequence of 40%, 60%, and 80% MVC. Each condition lasted 2 weeks and was performed 5 days per week, with a 2-week washout period between successive conditions.

During each exercise session, participants performed a 2-min isometric handgrip contraction with the right hand, followed by a 1-min rest period and then a 2-min isometric handgrip contraction with the left hand. This right-rest-left sequence was performed twice per day. Exercise was primarily performed as an unsupervised program. In participants undergoing HD, exercise performance was checked once weekly during dialysis.

Exercise safety was monitored throughout the intervention in accordance with established principles for exercise therapy in renal rehabilitation [16]. Participants were monitored for clinically relevant changes in vital signs and symptoms, and exercise was to be discontinued if the safety criteria requiring exercise termination were met.

#### 2.3.3. Outcome Measures

##### Primary Outcome: Peripheral Vascular Function

The primary clinical outcome was peripheral vascular function assessed using peripheral arterial tonometry (PAT). PAT measurements were performed using the EndoPAT 2000 system (Itamar Medical Ltd., Caesarea, Israel). Pneumatic finger probes were placed on both hands to continuously record digital pulse-wave amplitude. A blood-pressure cuff was placed on one upper arm to induce temporary arterial occlusion, while the contralateral arm served as the non-occluded control. In participants undergoing HD, arterial occlusion was performed only on the non-vascular-access arm; the vascular-access arm was not occluded and served as the contralateral control arm. Following cuff release, the change in digital pulse-wave amplitude during reactive hyperemia was recorded to assess the peripheral vascular response [17,18].

Measurements were performed according to the standardized EndoPAT procedure. For the present analysis, the unadjusted PAT ratio derived directly from the recorded pulse-wave amplitude response was used consistently across all participants and measurement conditions, rather than the automatically calculated reactive hyperemia index (RHI).

##### Inflammatory Markers

Serum IL-6 and TNF-α concentrations were assessed as inflammatory outcomes.

##### Muscle Strength

Handgrip strength was assessed as an upper-limb functional outcome. Lower-limb muscle strength was assessed as isometric knee-extension strength in the seated position using a GT-620 dynamometer (OG Wellness Technologies Co., Ltd., Okayama, Japan). Two measurements were obtained, and the maximum value was used for analysis. Lower-limb muscle strength was expressed in kilogram-force (kgf).

### 2.4. Statistical Analysis

Statistical analyses were performed using IBM SPSS Statistics version 29.0 (IBM Corp., Armonk, NY, USA).

#### 2.4.1. Experimental Study

End-of-study body weight, spontaneous physical activity, SBP, and forelimb grip strength were analyzed using two-way factorial models including CKD status (non-CKD vs. CKD), exercise exposure (non-exercise vs. exercise), and the CKD-by-exercise interaction.

Because IL-6 and TNF-α concentrations showed skewed distributions, these variables were natural log-transformed before inferential analysis, whereas untransformed concentrations are presented as medians [interquartile ranges] for descriptive purposes. Log-transformed cytokine concentrations were analyzed using the same two-factor framework.

Serum creatinine was measured before and after the experimental period and was analyzed using a linear mixed-effects model including time, CKD status, exercise exposure, and their interactions as fixed effects, with an animal-specific random intercept to account for repeated measurements.

#### 2.4.2. Clinical Study

Continuous variables were summarized as mean ± standard deviation (SD) or median [interquartile range (IQR)], as appropriate. Because IL-6 and TNF-α concentrations showed skewed distributions, these variables were natural log-transformed before inferential analysis.

Linear mixed-effects models were fitted separately for each clinical outcome. Group (HD vs. non-HD), exercise-intensity condition (rest, 40%, 60%, and 80% MVC), and the group-by-intensity interaction were included as fixed effects, with a participant-specific random intercept to account for repeated measurements. Exercise intensity was treated as a categorical variable. To facilitate interpretation of the magnitude and precision of the observed responses, exploratory within-group contrasts comparing each exercise-intensity condition with rest were additionally estimated with 95% confidence intervals. For PAT ratio, effects are presented as mean differences from rest. For IL-6 and TNF-α, which were analyzed after natural-log transformation, contrasts were back-transformed and are presented as ratios relative to rest with 95% confidence intervals. Because these contrasts were exploratory, no adjustment for multiple comparisons was applied, and emphasis was placed on the magnitude and precision of the estimates rather than on the statistical significance of individual contrasts.

Because the HD and non-HD groups were not age-matched and the total sample size was limited, extensive multivariable adjustment was avoided to minimize model overfitting. As an exploratory sensitivity analysis, age was added as a single covariate to models for the principal vascular and inflammatory outcomes. Between-group comparisons were considered exploratory, with emphasis placed on within-participant response patterns across exercise-intensity conditions. Given the small sample size, individual participant data were also visually examined to characterize response heterogeneity. No observations were excluded solely based on their numerical magnitude, and all available measurements were retained in the primary analyses.

All statistical analyses were independently verified using IBM SPSS Statistics, version 29.0 (IBM Corp., Armonk, NY, USA). All statistical tests were two-sided, and *p* < 0.05 was considered statistically significant.

### 2.5. Use of Generative Artificial Intelligence

During the preparation of this manuscript, the authors used ChatGPT (GPT-5; OpenAI, San Francisco, CA, USA) for English-language drafting and editorial assistance. The authors reviewed and edited all generated content and take full responsibility for the content of the publication.

## 3. Results

### 3.1. Experimental Study

#### 3.1.1. Body Weight and Spontaneous Physical Activity

At the end of the 4-week experimental period, body weight was significantly lower in the CKD animals than in the non-CKD animals (main effect of CKD, *p* < 0.001). No significant main effect of exercise (*p* = 0.896) or CKD-by-exercise interaction (*p* = 0.846) was observed. Mean body weights were 312.30 ± 12.07 g in the non-Ex group, 314.54 ± 5.26 g in the Ex group, 254.60 ± 15.96 g in the non-Ex CKD group, and 254.16 ± 22.08 g in the Ex CKD group.

Spontaneous physical activity was also lower in the CKD animals (main effect of CKD, *p* < 0.001). Activity was numerically higher in exercised animals, although the main effect of exercise did not reach statistical significance (*p* = 0.056), and no CKD-by-exercise interaction was observed (*p* = 0.679). Mean activity levels were 791.8 ± 137.7, 913.6 ± 73.2, 564.4 ± 132.0, and 644.8 ± 80.7-wheel counts/day in the non-Ex, Ex, non-Ex CKD, and Ex CKD groups, respectively.

#### 3.1.2. Forelimb Grip Strength and Systolic Blood Pressure

At study completion, forelimb grip strength was numerically higher in the exercised animals than in the non-exercised animals. Mean grip strength was 193.25 ± 4.65 g in the non-Ex group, 279.71 ± 84.92 g in the Ex group, 232.64 ± 35.99 g in the non-Ex CKD group, and 283.13 ± 113.86 g in the Ex CKD group. The main effect of exercise approached but did not reach statistical significance (*p* = 0.053). Neither the main effect of CKD (*p* = 0.523) nor the CKD-by-exercise interaction (*p* = 0.591) was significant.

End-of-study SBP did not significantly differ according to CKD status (*p* = 0.790) or exercise exposure (*p* = 0.259), and no significant CKD-by-exercise interaction was observed (*p* = 0.381). Mean SBP values were 133.0 ± 7.1, 134.2 ± 7.6, 127.8 ± 10.2, and 137.0 ± 13.5 mmHg in the non-Ex, Ex, non-Ex CKD, and Ex CKD groups, respectively.

#### 3.1.3. Serum Creatinine

Serum creatinine concentrations were higher in the CKD animals than in the non-CKD animals. Mean pre- and post-intervention serum creatinine concentrations were 0.228 ± 0.062 and 0.282 ± 0.131 mg/dL in the non-Ex group, 0.139 ± 0.032 and 0.220 ± 0.025 mg/dL in the Ex group, 0.520 ± 0.065 and 0.582 ± 0.043 mg/dL in the non-Ex CKD group, and 0.322 ± 0.078 and 0.388 ± 0.144 mg/dL in the Ex CKD group, respectively.

In the mixed-effects analysis, the temporal change in serum creatinine did not significantly differ according to exercise exposure (time-by-exercise interaction, *p* = 0.505), and no significant time-by-CKD-by-exercise interaction was observed (*p* = 0.694).

#### 3.1.4. Inflammatory Cytokines

Serum IL-6 concentrations differed significantly according to CKD status (main effect of CKD, *p* = 0.005), whereas neither the main effect of exercise (*p* = 0.249) nor the CKD-by-exercise interaction (*p* = 0.831) was significant. Median [IQR] IL-6 concentrations were 73.36 [72.36–94.53], 74.27 [72.22–91.10], 59.88 [58.69–61.07], and 56.14 [54.91–61.18] in the non-Ex, Ex, non-Ex CKD, and Ex CKD groups, respectively.

For TNF-α, no significant main effect of CKD (*p* = 0.420), exercise (*p* = 0.689), or CKD-by-exercise interaction (*p* = 0.898) was observed. Median [IQR] TNF-α concentrations were 9.40 [8.20–11.40], 11.93 [10.40–15.40], 5.24 [4.80–7.60], and 7.40 [5.40–9.60] in the non-Ex, Ex, non-Ex CKD, and Ex CKD groups, respectively (Table 1).

Serum creatinine was analyzed using a linear mixed-effects model including time, CKD status, exercise exposure, and their interactions.

#### 3.1.5. Renal Histopathology

**Figure 1:**
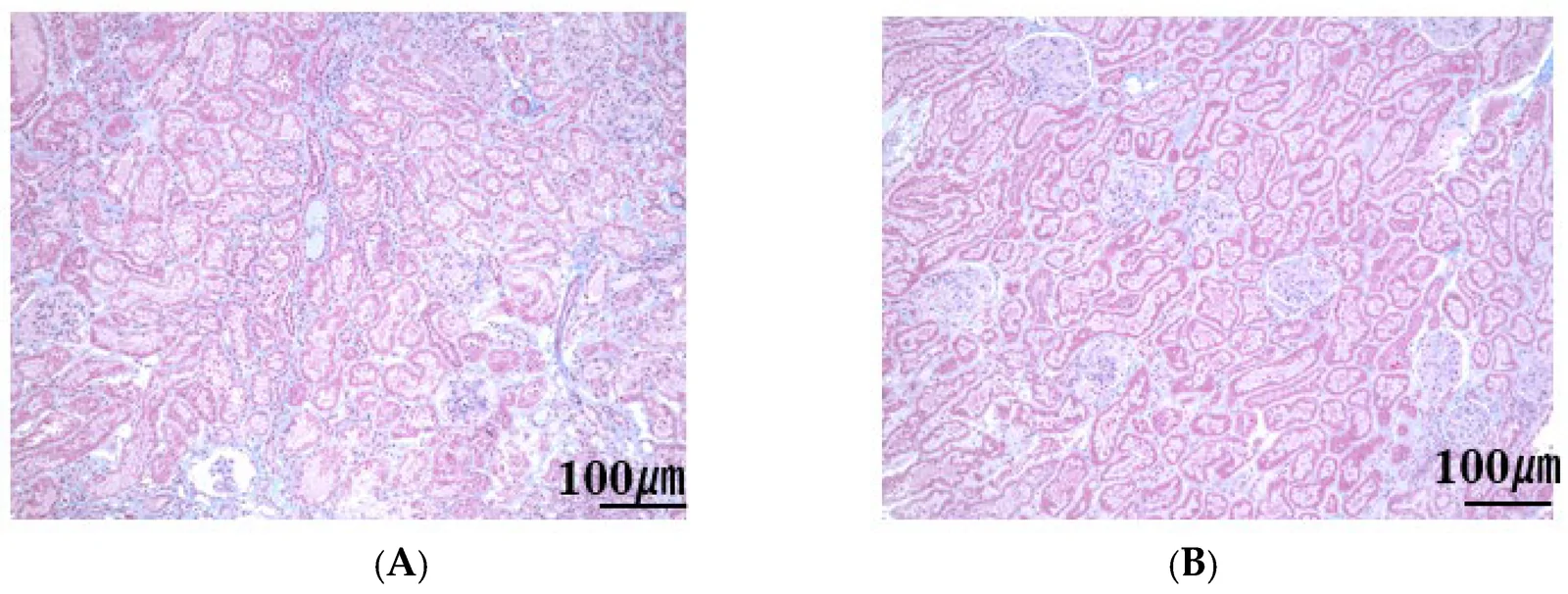
Representative renal histopathology in chronic kidney disease (CKD) rats with and without forelimb exercise. Representative Masson’s trichrome-stained kidney sections from (**A**) CKD/non-exercise and (**B**) CKD/exercise animals. No obvious exercise-associated histopathological aggravation was apparent on qualitative examination. Histopathological changes were not quantitatively assessed. Scale bars = 100 μm.

Representative Masson’s trichrome-stained kidney sections from CKD animals with and without forelimb exercise were examined qualitatively (Figure 1).

Overall, the animal experiment did not demonstrate significant exercise-associated differences in body weight, systolic blood pressure, serum inflammatory cytokines, or temporal changes in serum creatinine. Forelimb grip strength and spontaneous activity were numerically higher in the exercise groups, but these findings should be interpreted descriptively given the small group size (n = 5 per group). The animal findings were therefore considered complementary experimental observations rather than mechanistic evidence for the clinical vascular or inflammatory responses.

### 3.2. Clinical Study

#### 3.2.1. Participant Characteristics

Sixteen participants completed the clinical study, including eight patients undergoing maintenance HD and eight non-HD participants. The HD group was substantially older than the non-HD group (64.1 ± 17.0 vs. 31.6 ± 16.9 years). At rest, grip strength and lower-limb muscle strength were lower in the HD group, whereas circulating IL-6 and TNF-α concentrations were higher. Participant characteristics are summarized in Table 2.

Because the non-HD participants were not age-matched to the HD group, between-group comparisons were considered exploratory, and particular emphasis was placed on within-participant response patterns across exercise-intensity conditions.

#### 3.2.2. Peripheral Vascular Response

PAT ratio exhibited a significant group-by-intensity interaction (*p* = 0.0035), whereas neither the overall group effect (*p* = 0.959) nor the overall intensity effect (*p* = 0.400) was significant.

**Figure 2:**
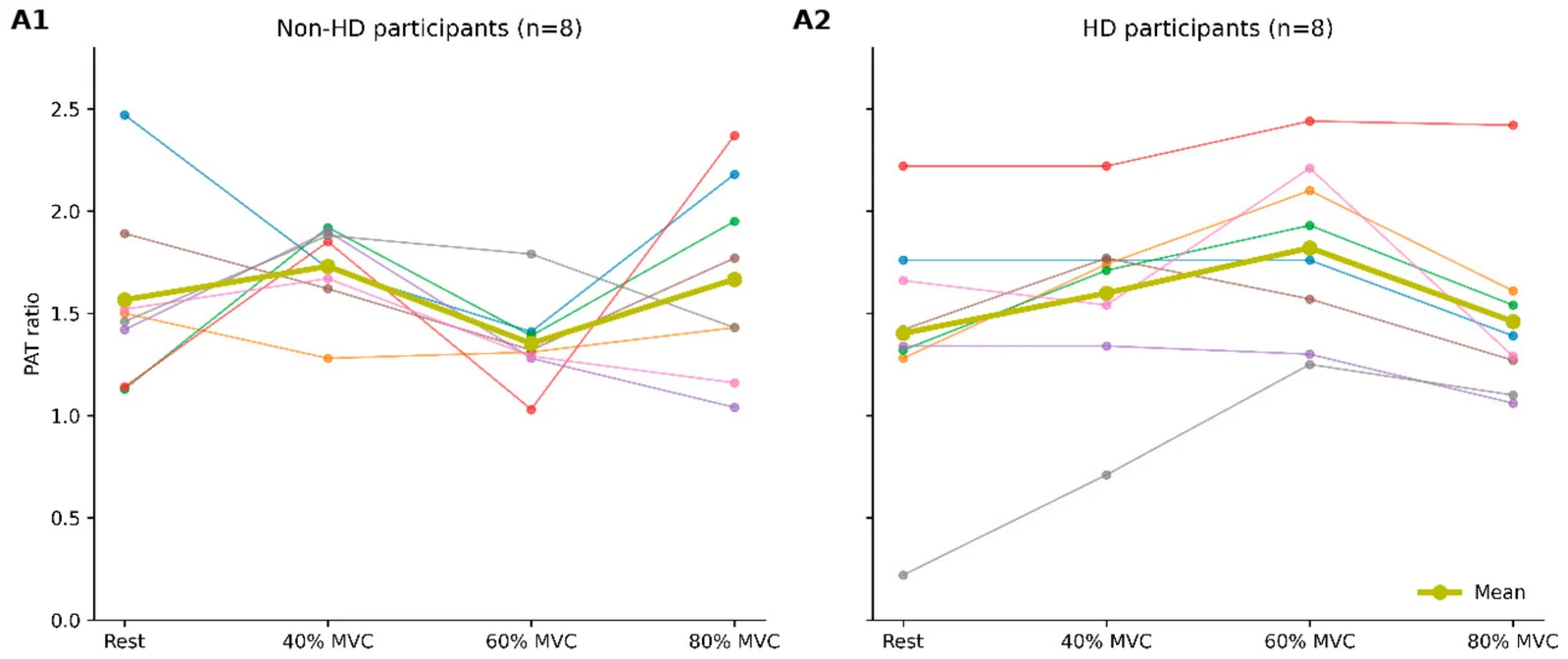

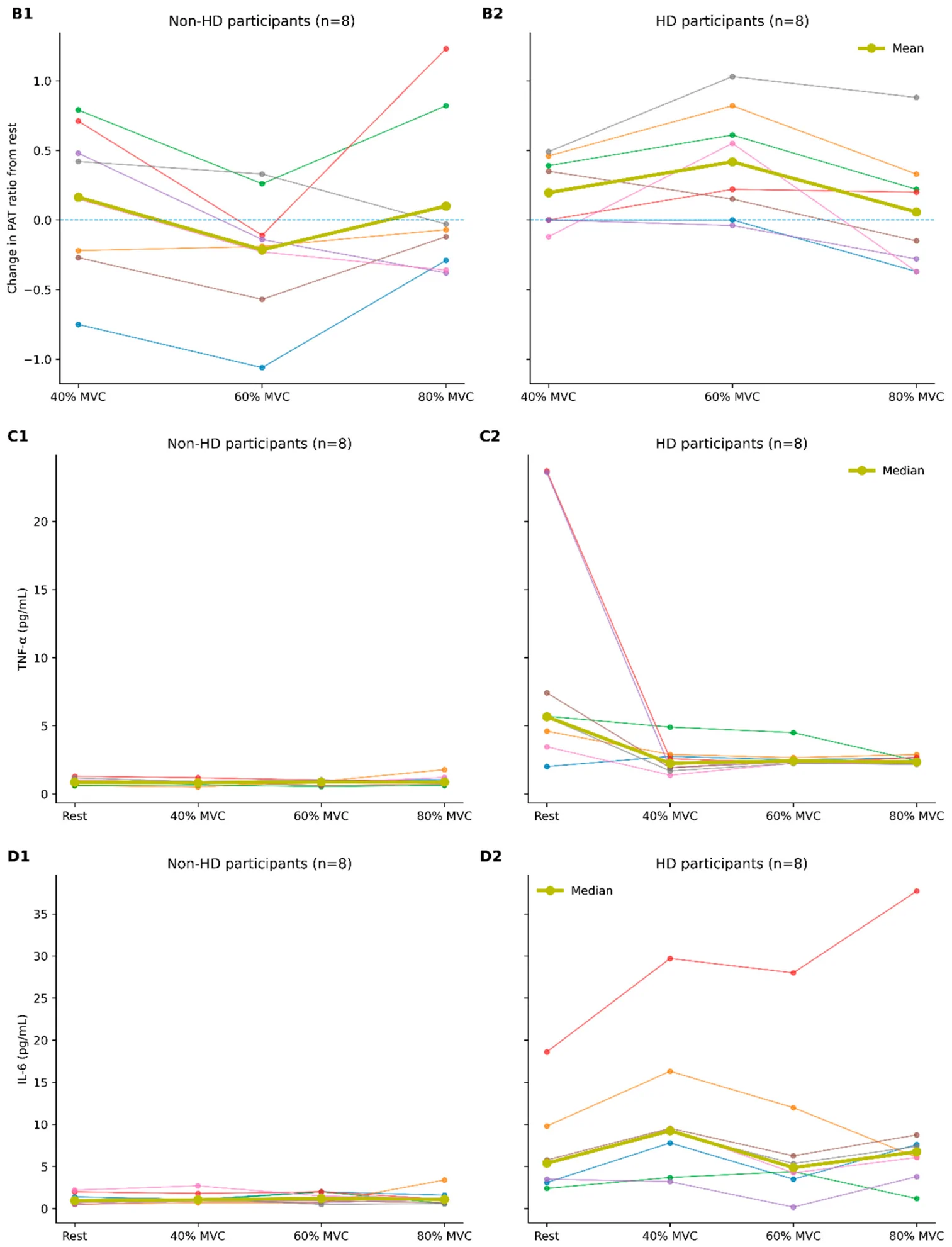
Vascular and inflammatory responses to different handgrip exercise conditions. (**A1**) PAT ratio in non-HD participants; (**A2**) PAT ratio in HD participants; (**B1**) change in PAT ratio from rest in non-HD participants; (**B2**) change in PAT ratio from rest in HD participants; (**C1**) TNF-α in non-HD participants; (**C2**) TNF-α in HD participants; (**D1**) IL-6 in non-HD participants; (**D2**) IL-6 in HD participants. PAT, peripheral arterial tonometry; TNF-α, tumor necrosis factor-α; IL-6, interleukin-6.

In the non-HD group, mean PAT ratios were 1.566 ± 0.436 at rest, 1.730 ± 0.214 at 40% MVC, 1.352 ± 0.211 at 60% MVC, and 1.666 ± 0.480 at 80% MVC. In the HD group, the corresponding values were 1.403 ± 0.572, 1.599 ± 0.437, 1.820 ± 0.429, and 1.460 ± 0.433, respectively (Figure 2A1,A2).

Mean changes in PAT ratio from rest in the HD group were +0.196 (95% CI, −0.012 to 0.404) at 40% MVC, +0.418 (95% CI, 0.088 to 0.747) at 60% MVC, and +0.058 (95% CI, −0.306 to 0.421) at 80% MVC. The largest mean numerical increase from rest was therefore observed following the 60% MVC condition; however, substantial interindividual variability was evident, and these exploratory contrasts should not be interpreted as establishing an optimal exercise intensity. Individual trajectories demonstrated substantial heterogeneity in PAT responses across conditions (Figure 2B1,B2), with some participants showing increases and others showing little change or decreases. All individual observations were retained in the analysis.

In an exploratory sensitivity analysis including age as a covariate, the group-by-intensity interaction for PAT ratio remained significant (*p* = 0.0035), whereas the age effect was not significant (*p* = 0.587).

#### 3.2.3. Inflammatory Responses

For log-transformed TNF-α, significant effects were observed for group, exercise intensity, and the group-by-intensity interaction (all *p* < 0.001). In the HD group, median [IQR] TNF-α concentrations were 5.665 [4.313–11.465] at rest, 2.245 [1.845–2.785] at 40% MVC, 2.420 [2.273–2.555] at 60% MVC, and 2.340 [2.223–2.655] at 80% MVC (Figure 2C2). In the HD group, TNF-α was lower under each exercise condition relative to rest. On the log-transformed scale, the corresponding back-transformed ratios relative to rest were 0.34 (95% CI, 0.15–0.78) at 40% MVC, 0.38 (95% CI, 0.18–0.83) at 60% MVC, and 0.36 (95% CI, 0.17–0.76) at 80% MVC. These estimates indicate lower TNF-α concentrations across the three exercise conditions compared with rest, but do not suggest a monotonic intensity-response pattern.

In the age-adjusted sensitivity analysis, the group-by-intensity interaction remained significant (*p* < 0.001), whereas age was not significant (*p* = 0.921).

For IL-6, the back-transformed ratios relative to rest in the HD group were 1.61 (95% CI, 1.28–2.03) at 40% MVC, 0.80 (95% CI, 0.31–2.07) at 60% MVC, and 1.19 (95% CI, 0.75–1.87) at 80% MVC. However, neither the overall effect of exercise condition nor the group-by-condition interaction was statistically significant, and no consistent response pattern across the exercise conditions was evident (Figure 2D2). The group-by-intensity interaction remained non-significant after exploratory adjustment for age (*p* = 0.396; age effect, *p* = 0.326).

#### 3.2.4. Muscle Strength

Grip strength differed significantly between groups (*p* < 0.001), but neither the overall intensity effect (*p* = 0.759) nor the group-by-intensity interaction (*p* = 0.924) was significant.

Lower-limb muscle strength also differed significantly between groups (*p* < 0.001), whereas neither the intensity effect (*p* = 0.447) nor the group-by-intensity interaction (*p* = 0.940) was significant. Thus, no consistent changes across exercise-intensity conditions in either upper- or lower-limb muscle strength were detected during the relatively short intervention periods (Table 3).

#### 3.2.5. Adverse Events

All participants completed the planned exercise conditions. No participant required exercise termination because of exercise-related blood pressure changes or other clinically relevant safety concerns. No cardiovascular events, musculoskeletal injuries, falls, or other adverse events considered attributable to the exercise intervention were observed.

## 4. Discussion

The principal finding of the present study was that vascular responses to handgrip exercise differed across exercise-intensity conditions between patients undergoing HD and non-HD participants. A significant group-by-intensity interaction was observed for PAT ratio, and among the patients undergoing HD, the largest mean numerical increase from rest was observed following the 60% MVC condition. However, this response was not monotonic across exercise intensities, and substantial interindividual variability was evident. TNF-α also showed a significant group-by-intensity interaction, whereas IL-6 and muscle strength did not exhibit comparable response patterns across exercise-intensity conditions. In the complementary experimental study, repeated forelimb exercise was not associated with significant differences in body weight, SBP, serum creatinine trajectories, or circulating IL-6 and TNF-α, while spontaneous activity and forelimb grip strength were numerically higher in exercised animals. Taken together, these findings suggest that responses to localized resistance exercise were heterogeneous across the conditions studied and differed according to the outcome assessed.

### 4.1. Vascular Response Patterns Across Handgrip Exercise Conditions

The significant group-by-intensity interaction for PAT ratio represents the primary clinical finding of this study. PAT provides a non-invasive assessment of digital reactive hyperemia and has been applied to the evaluation of peripheral vascular function in patients undergoing maintenance HD [17,18]. In patients undergoing HD, the mean change in PAT ratio from rest was +0.196 (95% CI, −0.012 to 0.404) at 40% MVC, +0.418 (95% CI, 0.088 to 0.747) at 60% MVC, and +0.058 (95% CI, −0.306 to 0.421) at 80% MVC. Thus, the largest numerical vascular response occurred following the 60% MVC condition, rather than increasing progressively with exercise intensity. Repeated isometric handgrip training has previously been associated with improvements in resting endothelium-dependent vasodilation in populations with vascular dysfunction [19]. Importantly, these findings should not be interpreted as demonstrating that 60% MVC is an optimal exercise intensity or that 80% MVC is detrimental. Previous handgrip training studies have also demonstrated vascular adaptations using moderate contraction intensities, including 60% of maximal handgrip strength in older adults [11]. The study was not designed or powered to establish an optimal dose, and the individual responses showed considerable variability.

The observed non-linear response is physiologically plausible. Previous work demonstrates that handgrip intensity can modify shear and vascular responses [12]. Handgrip exercise increases local metabolic demand and can augment blood flow and shear stress, which are important stimuli for endothelial nitric oxide production and vascular adaptation [8,9]. However, vascular responses to increasing contraction intensity are complex, and a greater contractile intensity does not necessarily translate into a proportionally greater endothelial stimulus [12,20]. This complex relationship between exercise intensity, shear stress, and vascular responsiveness may partly explain why the largest mean PAT response in the HD group occurred at an intermediate rather than the highest intensity.

Such variation across exercise-intensity conditions may be particularly relevant for future investigation in HD populations. Previous meta-analytic evidence also suggests that the relationship between resistance-exercise intensity and endothelial adaptation may not be simply linear [21]. Patients undergoing HD frequently exhibit endothelial dysfunction associated with uremia, oxidative stress, chronic inflammation [1,3,4], vascular calcification, and reduced physical activity. Their vascular reserve and responsiveness to mechanical stimuli may therefore differ from those of individuals without HD. The contrasting PAT response patterns observed between the HD and non-HD groups are consistent with this possibility. Previous evidence in CKD also indicates that exercise effects on vascular outcomes may vary according to exercise characteristics and the vascular endpoint examined [7]. Nevertheless, because the two groups were substantially different in age, direct between-group physiological comparisons require caution.

The age-adjusted sensitivity analysis is informative in this regard. The group-by-intensity interaction for PAT ratio remained significant after the inclusion of age as a covariate, whereas age itself was not significant. This suggests that the observed interaction was not simply eliminated by statistical adjustment for age. However, given the small sample size and marked age imbalance, covariate adjustment cannot fully compensate for the lack of age matching. The individual trajectories further demonstrated considerable heterogeneity in vascular responses, underscoring that the group mean should not be interpreted as representing a uniform response among patients undergoing HD. Accordingly, the present results should be regarded as hypothesis-generating evidence of differential vascular responsiveness rather than definitive evidence of a disease-specific effect.

### 4.2. Inflammatory Responses Were Marker-Specific

Another notable finding was the difference between the responses of TNF-α and IL-6. TNF-α showed significant effects of group, exercise intensity, and their interaction. Among patients undergoing HD, TNF-α concentrations were lower under all three exercise-intensity conditions than at rest, but there was no clear monotonic dose–response relationship. In contrast, IL-6 showed a strong group difference but no significant intensity effect or group-by-intensity interaction.

These findings argue against interpreting the biological effects of handgrip exercise as a uniform anti-inflammatory response. Exercise-related cytokine responses are complex and may depend on exercise modality, contraction intensity, duration, training status, underlying disease, and the timing of blood sampling [13,14]. Recent systematic reviews and meta-analytic evidence in patients undergoing HD have reported heterogeneous effects of exercise on inflammatory markers, including CRP, IL-6, and TNF-α, with substantial variability in interventions and overall certainty of evidence [14,20]. Moreover, IL-6 and TNF-α have distinct biological roles and may not respond in parallel [13,22]. The absence of a significant IL-6 interaction despite the TNF-α response therefore does not necessarily represent an inconsistent result; rather, it may reflect marker-specific responses to localized exercise. TNF-α concentrations were lower under all three exercise conditions than at rest in the HD group, whereas no monotonic pattern across increasing exercise intensities was evident. Thus, these findings should not be interpreted as demonstrating an intensity-dependent anti-inflammatory effect of handgrip exercise. Because the exercise conditions were administered in a fixed sequence, the observed TNF-α pattern may reflect not only the exercise condition itself but also period, training, or carryover effects that cannot be separated in the present design. In addition, the small sample size and substantial between-participant variability warrant cautious interpretation. These findings therefore provide exploratory evidence of changes in TNF-α across the study conditions rather than evidence for a specific anti-inflammatory dose–response relationship.

However, because the experimental and clinical components differed substantially in exercise protocols, study design, and outcome assessment, the animal cytokine findings should not be interpreted as mechanistic confirmation of the clinical TNF-α response pattern. Rather, the two components provide separate observations regarding inflammatory responses to localized forelimb exercise under their respective experimental and clinical conditions.

### 4.3. Functional and Physiological Findings from the Experimental Study

The experimental study was intended to provide complementary physiological information rather than direct evidence of clinical efficacy. CKD animals had lower final body weight and spontaneous physical activity, consistent with the systemic consequences of experimental kidney disease. Forelimb grip strength and spontaneous activity were numerically higher in the exercised animals, although the exercise main effects did not reach conventional statistical significance. These observations may indicate a functional response to repeated forelimb exercise, but the small number of animals and the absence of baseline grip-strength measurements preclude conclusions regarding training-induced improvement.

No significant exercise-related differences were detected in final body weight or SBP. Similarly, the temporal change in serum creatinine did not differ significantly according to exercise exposure, including when CKD status was considered. However, baseline serum creatinine was numerically lower in the CKD exercise group than in the CKD non-exercise group (0.322 ± 0.078 vs. 0.520 ± 0.065 mg/dL), indicating baseline imbalance in renal function between these small groups. Accordingly, the absence of a significant time-by-exercise interaction should be interpreted cautiously and does not establish the absence of an exercise effect on renal function. These findings do not demonstrate renal protection or deterioration attributable to exercise.

Representative Masson’s trichrome-stained kidney sections likewise showed no obvious exercise-associated histopathological aggravation on qualitative examination. However, because renal histopathology was not quantitatively assessed, these observations should be regarded as descriptive only. Together, these experimental findings revealed no clear adverse exercise-related differences in the measured physiological, renal, or inflammatory outcomes under the conditions tested, while providing no evidence for renal-protective or generalized anti-inflammatory effects.

### 4.4. Muscle Strength and Training Specificity

Neither grip strength nor lower-limb muscle strength showed a significant intensity effect or group-by-intensity interaction in the clinical study. Several factors may account for these findings. First, each exercise-intensity condition lasted only 2 weeks, which is relatively short for the development of measurable changes in muscle strength. Early responses to resistance exercise may involve neural adaptations, whereas substantial structural muscular adaptation generally requires a longer training period [23].

Second, the intervention specifically targeted the forearm musculature. The absence of a consistent change in lower-limb muscle strength is therefore compatible with the principle of training specificity [23] and should not be interpreted as evidence that the intervention was ineffective. Rather, the primary purpose of the present protocol was to investigate vascular and inflammatory responses to different handgrip intensities, not to provide a comprehensive whole-body resistance-training stimulus.

These findings also reinforce the distinction between an acute or short-term physiological response and longer-term functional adaptation. The observed PAT responses may occur on a different timescale from measurable changes in muscle performance. Longer intervention studies will be required to determine whether repeated handgrip exercise produces sustained vascular adaptation or clinically meaningful improvements in physical function.

### 4.5. Clinical Implications

The present findings suggest that a higher contraction intensity did not consistently correspond to a larger vascular response under the conditions studied. However, the present study was not designed to identify a clinically optimal exercise intensity.

From a clinical perspective, the present findings should not be used to recommend a specific handgrip exercise intensity for patients undergoing HD. Although the largest mean numerical increase in PAT ratio from rest was observed following the 60% MVC condition, substantial interindividual variability was present, and the fixed intervention sequence prevents the separation of exercise-condition effects from potential period, training, or carryover effects. The findings instead suggest that vascular and inflammatory responses to localized handgrip exercise may vary among patients and across exercise conditions. Future adequately powered randomized studies with counterbalanced or randomized exercise-intensity sequences are needed to determine whether specific intensities produce reproducible vascular benefits and to identify patient characteristics associated with individual response patterns.

This concept may be particularly relevant to HD populations in whom age, dialysis vintage, cardiovascular comorbidity, vascular calcification, autonomic dysfunction, and physical capacity vary considerably. Future studies should therefore examine predictors of vascular responsiveness and determine whether individualized intensity selection can improve the efficacy and tolerability of localized resistance exercise.

### 4.6. Strengths and Limitations

This study has several strengths. First, vascular responses were examined across three predefined handgrip exercise intensities within the same participants, allowing the characterization of individual response patterns across exercise-intensity conditions. Second, vascular, inflammatory, and functional outcomes were assessed concurrently. Third, the complementary experimental study provided additional physiological information regarding repeated localized forelimb exercise in the presence and absence of CKD.

Several limitations should also be acknowledged. First, the clinical sample was small, with eight participants in each group, and the findings should therefore be considered exploratory. Second, the HD and non-HD groups were not age matched. Although age-adjusted sensitivity analyses yielded similar results for the principal vascular and inflammatory interactions, statistical adjustment cannot eliminate residual confounding arising from the substantial age difference.

Third, the exercise-intensity conditions were performed in a fixed sequence of 40%, 60%, and 80% MVC rather than in randomized order. Potential period, carryover, or order effects therefore cannot be completely excluded despite the 2-week washout intervals. Furthermore, although washout periods were incorporated into the protocol, measurements obtained at the end of each washout period were not available for the present analysis; consequently, a complete return to the baseline between conditions could not be statistically verified.

Fourth, each intensity condition lasted only 2 weeks. This duration may have been sufficient to detect short-term vascular or inflammatory responses but was likely insufficient to evaluate longer-term muscular adaptation or sustained cardiovascular benefit. Fifth, the non-HD group should be regarded as a reference group rather than a matched control group, and direct disease-specific comparisons should therefore be interpreted cautiously.

Finally, the experimental study also had a small sample size. Several outcomes were available only at the end of the experimental period, limiting the evaluation of within-animal changes. Renal histopathology was assessed qualitatively rather than quantitatively, and the animal findings therefore provide complementary biological context rather than mechanistic proof of the clinical observations.

### 4.7. Future Directions

Larger randomized studies are needed to determine whether the vascular response patterns observed across exercise-intensity conditions in this exploratory study are reproducible. Future trials should include age-matched comparison groups, randomize the order of exercise intensities, and obtain measurements before and after each intervention and washout period. Longer intervention periods would also permit an evaluation of whether short-term changes in vascular responsiveness translate into sustained improvements in endothelial function, physical performance, or cardiovascular outcomes.

It may also be useful to investigate determinants of individual responsiveness. Given the marked variability in PAT responses observed among patients undergoing HD, characteristics such as baseline endothelial function, age, dialysis vintage, cardiovascular disease, inflammatory status, and physical capacity may modify the response to handgrip exercise. Such work could ultimately contribute to more individualized exercise prescription in HD.

## 5. Conclusions

Heterogeneous vascular and inflammatory response patterns were observed across the fixed-sequence handgrip exercise conditions. In patients undergoing HD, the largest mean numerical increase in PAT ratio from rest was observed following the 60% MVC condition; however, substantial interindividual variability was present. Given the small sample size, fixed intervention sequence, and differences in baseline characteristics between the HD and non-HD groups, these findings should not be interpreted as demonstrating causal intensity-dependent or HD-specific effects. Rather, they provide hypothesis-generating observations for future randomized studies of individualized handgrip exercise in HD populations.

## Figures and Tables

**Table 1:** Experimental findings according to CKD status and exercise exposure.

**Outcome**	**Non-Ex**	**Ex**	**Non-Ex CKD**	**Ex CKD**	**CKD *p***	**Exercise *p***		**Interaction *p***
Body weight, g	312.30 ± 12.07	314.54 ± 5.26	254.60 ± 15.96	254.16 ± 22.08	<0.001	0.896		0.846
Activity, wheel/day	791.8 ± 137.7	913.6 ± 73.2	564.4 ± 132.0	644.8 ± 80.7	<0.001	0.056		0.679
Forelimb grip strength, g	193.25 ± 4.65	279.71 ± 84.92	232.64 ± 35.99	283.13 ± 113.86	0.523	0.053		0.591
SBP, mmHg	133.0 ± 7.1	134.2 ± 7.6	127.8 ± 10.2	137.0 ± 13.5	0.790	0.259		0.381
**Outcome**	**non-Ex**	**Ex**	**non-Ex CKD**	**Ex CKD**	**CKD *p***	**Exercise *p***		**Interaction *p***
IL-6	73.36 [72.36–94.53]	74.27 [72.22–91.10]	59.88 [58.69–61.07]	56.14 [54.91–61.18]	0.005	0.249		0.831
TNF-α	9.40 [8.20–11.40]	11.93 [10.40–15.40]	5.24 [4.80–7.60]	7.40 [5.40–9.60]	0.420	0.689		0.898
Group			Pre, mg/dL	Post, mg/dL	ΔCr			
non-Ex			0.228 ± 0.062		0.282 ± 0.131		+0.054 ± 0.076	
Ex			0.139 ± 0.032		0.220 ± 0.025		+0.081 ± 0.041	
non-Ex CKD			0.520 ± 0.065		0.582 ± 0.043		+0.062 ± 0.059	
Ex CKD			0.322 ± 0.078		0.388 ± 0.144		+0.067 ± 0.100	

Values are presented as mean ± SD or median [IQR], as appropriate. IL-6 and TNF-α were natural log-transformed before inferential analysis; untransformed values are presented for descriptive purposes. End-of-study outcomes were analyzed using two-way factorial models including CKD status, exercise exposure, and their interaction. CKD, chronic kidney disease; Ex, exercise; SBP, systolic blood pressure; IL-6, interleukin-6; TNF-α, tumor necrosis factor-α; Cr, creatinine; SD, standard deviation; IQR, interquartile range.

**Table 2:** Characteristics of the study participants.

Characteristic	Non-HD (n = 8)	HD (n = 8)
Male sex, n (%)	7 (87.5)	5 (62.5)
Age, years	31.6 ± 16.9	64.1 ± 17.0
Body weight, kg	59.6 ± 2.2	63.7 ± 8.0
Height, cm	170.5 ± 3.5	159.5 ± 4.5
BMI, kg/m^2^	20.5 ± 1.0	25.1 ± 3.1
Grip strength, kg	40.46 ± 5.51	23.54 ± 6.04
Lower-limb muscle strength, kgf	47.96 ± 8.40	14.66 ± 6.40
PAT ratio	1.566 ± 0.436	1.403 ± 0.572
IL-6, pg/mL	0.95 [0.65–1.55]	5.39 [3.40–6.78]
TNF-α, pg/mL	0.855 [0.713–1.058]	5.665 [4.313–11.465]
Pre-exercise systolic BP, mmHg	—	130.0 ± 24.1
Pre-exercise diastolic BP, mmHg	—	76.0 ± 10.4
Dialysis vintage, months	—	94.1 ± 16.5
Diabetes mellitus, n (%)	—	3 (37.5)
Antihyperglycemic medication, n (%)	—	3 (37.5)
Antihypertensive medication, n (%)	—	4 (50.0)

Values are presented as mean ± SD or median [IQR], as appropriate. BMI, body mass index; HD, hemodialysis; PAT, peripheral arterial tonometry; IL-6, interleukin-6; TNF-α, tumor necrosis factor-α; BP, blood pressure; SD, standard deviation; IQR, interquartile range.

**Table 3:** Vascular, inflammatory, and physical responses across the handgrip exercise-intensity conditions.

Outcome	Group	Rest	40% MVC	60% MVC	80% MVC	Group *p*	Intensity *p*	Group × Intensity *p*
PAT ratio	non-HD	1.566 ± 0.436	1.730 ± 0.214	1.352 ± 0.211	1.666 ± 0.480	0.959	0.400	0.0035
	HD	1.403 ± 0.572	1.599 ± 0.437	1.820 ± 0.429	1.460 ± 0.433			
IL-6, pg/mL	non-HD	0.95 [0.65–1.55]	1.05 [0.98–1.35]	1.20 [0.70–2.00]	1.10 [0.68–1.30]	<0.001	0.199	0.396
	HD	5.39 [3.40–6.78]	9.25 [6.78–11.22]	4.88 [4.06–7.70]	6.75 [5.50–7.89]			
TNF-α, pg/mL	non-HD	0.855 [0.713–1.058]	0.810 [0.685–0.878]	0.870 [0.758–0.965]	0.865 [0.770–1.088]	<0.001	<0.001	<0.001
	HD	5.665 [4.313–11.465]	2.245 [1.845–2.785]	2.420 [2.273–2.555]	2.340 [2.223–2.655]			
Grip strength, kg	non-HD	40.46 ± 5.51	40.84 ± 6.34	41.81 ± 6.45	40.71 ± 6.64	<0.001	0.759	0.924
	HD	23.54 ± 6.04	24.23 ± 8.27	24.53 ± 6.03	24.91 ± 7.09			
Lower-limb muscle strength, kgf	non-HD	47.96 ± 8.40	51.95 ± 15.78	47.89 ± 21.14	50.00 ± 21.47	<0.001	0.447	0.940
	HD	14.66 ± 6.40	17.37 ± 5.83	14.93 ± 4.52	14.41 ± 4.91			

Values are presented as mean ± SD or median [IQR], as appropriate. IL-6 and TNF-α were natural log-transformed before inferential analysis because of their skewed distributions; untransformed values are presented for descriptive purposes. *p*-values for the fixed effects of group, exercise intensity, and the group-by-intensity interaction were obtained from linear mixed-effects models with participant-specific random intercepts. HD, hemodialysis; MVC, maximal voluntary contraction; PAT, peripheral arterial tonometry; IL-6, interleukin-6; TNF-α, tumor necrosis factor-α. SD, standard deviation; IQR, interquartile range.

## Data Availability

The data supporting the findings of this study are available from the corresponding author upon reasonable request. The clinical data are not publicly available because of the privacy and ethical considerations related to the study participants.

## References

[1] Cho M.E., Brunt V.E., Shiu Y.T., Bunsawat K. (2025). Endothelial dysfunction in chronic kidney disease: A clinical perspective. Am. J. Physiol. Heart Circ. Physiol..

[2] Gomes Neto M., de Lacerda F.F.R., Lopes A.A., Martinez B.P., Saquetto M.B. (2018). Intradialytic exercise training modalities on physical functioning and health-related quality of life in patients undergoing maintenance hemodialysis: Systematic review and meta-analysis. Clin. Rehabil..

[3] Roumeliotis S., Mallamaci F., Zoccali C. (2020). Endothelial Dysfunction in Chronic Kidney Disease, from Biology to Clinical Outcomes: A 2020 Update. J. Clin. Med..

[4] Kirkman D.L., Chavez D.A. (2024). Exercise for chronic kidney disease: Effects on vascular and cardiopulmonary function. Am. J. Physiol.-Heart Circ. Physiol..

[5] Zhao S., Zhong G., Lv A., Tao Y., Liu H., Lv H., Zhai Z., Li Y., Hua Y., Kang Y. (2026). Intradialytic Exercise Interventions to Enhance Physical Activity Levels in Hemodialysis Patients: A Systematic Review and Meta-Analysis. Am. J. Nephrol..

[6] Verrelli D., Sharma A., Alexiuk J., Tays Q., Rossum K., Sharma M., Ford E., Iansavitchene A., Al-Jaishi A.A., Whitlock R. (2024). Effect of Intradialytic Exercise on Cardiovascular Outcomes in Maintenance Hemodialysis: A Systematic Review and Meta-Analysis. Kidney360.

[7] Davies M.D., Hughes F., Sandoo A., Alejmi A., Macdonald J.H. (2023). The effect of exercise on vascular health in chronic kidney disease: A systematic review and meta-analysis of randomized controlled trials. Am. J. Physiol. Ren. Physiol..

[8] Ashor A.W., Lara J., Siervo M., Celis-Morales C., Oggioni C., Jakovljevic D.G., Mathers J.C. (2015). Exercise modalities and endothelial function: A systematic review and dose-response meta-analysis of randomized controlled trials. Sports Med..

[9] Tinken T.M., Thijssen D.H., Hopkins N., Dawson E.A., Cable N.T., Green D.J. (2010). Shear stress mediates endothelial adaptations to exercise training in humans. Hypertension.

[10] Farah B.Q., Germano-Soares A.H., Rodrigues S.L.C., Santos C.X., Barbosa S.S., Vianna L.C., Cornelissen V.A., Ritti-Dias R.M. (2017). Acute and Chronic Effects of Isometric Handgrip Exercise on Cardiovascular Variables in Hypertensive Patients: A Systematic Review. Sports.

[11] Dobrosielski D.A., Greenway F.L., Welsh D.A., Jazwinski S.M., Welsch M.A. (2009). Modification of vascular function after handgrip exercise training in 73- to 90-yr-old men. Med. Sci. Sports Exerc..

[12] Atkinson C.L., Carter H.H., Dawson E.A., Naylor L.H., Thijssen D.H., Green D.J. (2015). Impact of handgrip exercise intensity on brachial artery flow-mediated dilation. Eur. J. Appl. Physiol..

[13] Pedersen B.K., Steensberg A., Fischer C., Keller C., Ostrowski K., Schjerling P. (2001). Exercise and cytokines with particular focus on muscle-derived IL-6. Exerc. Immunol. Rev..

[14] Meléndez Oliva E., Villafañe J.H., Alonso Pérez J.L., Alonso Sal A., Molinero Carlier G., Quevedo García A., Turroni S., Martínez-Pozas O., Valcárcel Izquierdo N., Sánchez Romero E.A. (2022). Effect of Exercise on Inflammation in Hemodialysis Patients: A Systematic Review. J. Pers. Med..

[15] Watanabe Y., Kawanishi H., Suzuki K., Nakai S., Tsuchida K., Tabei K., Akiba T., Masakane I., Takemoto Y., Tomo T. (2015). Japanese Society for Dialysis Therapy clinical guideline for “Maintenance Hemodialysis: Hemodialysis Prescriptions”. Ther. Apher. Dial..

[16] Yamagata K., Hoshino J., Sugiyama H., Hanafusa N., Shibagaki Y., Komatsu Y., Konta T., Fujii N., Kanda E., Sofue T. (2019). Clinical practice guideline for renal rehabilitation: Systematic reviews and recommendations of exercise therapies in patients with kidney diseases. Ren. Replace. Ther..

[17] Liu W., Meng M., Chen J., Wang L., Sun Z., Li X., Zhou J., Gao C., Zhou J., Chu H. (2017). Reactive Hyperemia Index in Patients on Maintenance Hemodialysis: Cross-sectional Data from a Cohort Study. Sci. Rep..

[18] Bonetti P.O., Pumper G.M., Higano S.T., Holmes D.R., Kuvin J.T., Lerman A. (2004). Noninvasive identification of patients with early coronary atherosclerosis by assessment of digital reactive hyperemia. J. Am. Coll. Cardiol..

[19] McGowan C.L., Levy A.S., Millar P.J., Guzman J.C., Morillo C.A., McCartney N., Macdonald M.J. (2006). Acute vascular responses to isometric handgrip exercise and effects of training in persons medicated for hypertension. Am. J. Physiol. Heart Circ. Physiol..

[20] Zhang K., Wang C., Zhang N., Teng Z., Wang R., Wei S., Ni C. (2025). Effect of different exercise types on level of inflammation in hemodialysis patients: A systematic review and network meta-analysis. Complement. Ther. Clin. Pract..

[21] Zhang Y., Zhang Y.J., Zhang H.W., Ye W.B., Korivi M. (2021). Low-to-Moderate-Intensity Resistance Exercise Is More Effective than High-Intensity at Improving Endothelial Function in Adults: A Systematic Review and Meta-Analysis. Int. J. Environ. Res. Public Health.

[22] Pedersen B.K., Steensberg A., Schjerling P. (2001). Exercise and interleukin-6. Curr. Opin. Hematol..

[23] Del Vecchio A., Enoka R.M., Farina D. (2024). Specificity of early motor unit adaptations with resistive exercise training. J. Physiol..

